# Long‐Term, Site‐Specific Effectiveness of Tralokinumab in Atopic Dermatitis: A 72‐Week Real‐World Study

**DOI:** 10.1111/1346-8138.70138

**Published:** 2026-01-05

**Authors:** Mizuki Shiba, Teppei Hagino, Akihiko Uchiyama, Hidehisa Saeki, Eita Fujimoto, Sei‐ichiro Motegi, Naoko Kanda

**Affiliations:** ^1^ Department of Dermatology Nippon Medical School Chiba Hokusoh Hospital Inzai Japan; ^2^ Department of Dermatology Gunma University Graduate School of Medicine Maebashi Japan; ^3^ Department of Dermatology Nippon Medical School Tokyo Japan; ^4^ Department of Dermatology Fujimoto Dermatology Clinic Funabashi Japan

**Keywords:** atopic dermatitis, interleukin‐13, real‐world, site‐specific, tralokinumab

## Abstract

Tralokinumab, an anti‐IL‐13 antibody, is effective for atopic dermatitis (AD); however, its long‐term (> 1 year) effectiveness specific to each anatomical site is unknown in real‐world settings. To evaluate 72‐week effectiveness of tralokinumab on different anatomical sites in AD, we studied 208 patients with moderate‐to‐severe AD treated with tralokinumab for 72 weeks. Eczema area and severity index (EASI) scores were analyzed on four anatomical sites (head/neck, trunk, upper limbs, and lower limbs). Tralokinumab consistently reduced EASI on all anatomical sites. The achievement rates of EASI 75 and 100 on each site gradually increased from Week 4 to Week 72, and those on lower limbs appeared higher compared to the other sites. The percent reductions of EASI throughout 72 weeks appeared slightly lower on head and neck compared to the other sites. Week 72 achievement rate of EASI 75 on head/neck, trunk, upper limbs, or lower limbs was 80.4%, 80%, 80.3%, or 86.7%, while that of EASI 100 was 37.3%, 25.0%, 27.4%, and 40.0%, respectively. Tralokinumab reduced EASI scores through 72 weeks across different anatomical sites in AD patients. The achievement rates of EASI 75 and 100 appeared slightly higher on lower limbs compared to the other sites.

## 1 Introduction

Atopic dermatitis (AD) is a chronic inflammatory skin disease characterized by type 2‐skewed inflammation, skin barrier dysfunction, severe itching, and skin dysbiosis [1, 2, 3]. Recurrent eczematous lesions and intense itch in AD are known to have a serious impact on patients' quality of life (QoL). They cause sleep disturbance, psychological stress, and pain, and lead to a major disease burden. Multiple genetic or environmental factors contribute to the pathogenesis of AD and cause skin barrier disruption, immune dysregulation, or dysbiosis [4, 5]. AD is driven by various cytokines, including interleukin (IL)‐4, IL‐5, IL‐13, IL‐31, IL‐22, and thymic stromal lymphopoietin. Among these, IL‐13 plays a major role in the development of AD and contributes to skin barrier dysfunction, inflammation, and severe itch, including decreased epidermal filaggrin expression. Prior studies also showed that IL‐13 is one of the most prominent type 2 cytokines expressed in the lesional skin of patients with AD [6, 7, 8, 9, 10, 11, 12]. Conventional systemic therapies for moderate‐to‐severe AD, such as cyclosporine and systemic corticosteroids, often cause insufficient efficacy and safety concerns. There has been a strong need to develop new treatments that target the main inflammatory pathways in AD. The introduction of dupilumab, an antibody to IL‐4 receptor α that blocks signaling of both IL‐4 and IL‐13, showed remarkable efficacy in moderate‐to‐severe AD [13]. Based on this success, specific monoclonal antibodies that directly inhibit IL‐13 have been developed. Tralokinumab is a fully human immunoglobulin (Ig)G4 monoclonal antibody that specifically neutralizes IL‐13 [14, 15, 16, 17]. It has been approved in many countries for moderate‐to‐severe AD. Phase 3 clinical trials for tralokinumab treatment showed significant improvement of skin symptoms together with high tolerability and safety [18, 19, 20, 21, 22]. Real‐world effectiveness and safety of tralokinumab for AD have also been reported [23, 24, 25, 26, 27, 28, 29]. Furthermore, tralokinumab is effective even in patients who previously received systemic therapy [30]. However, long‐term (> 1 year) real‐world data that evaluate effectiveness by individual anatomical sites remain limited.

The effects of tralokinumab may vary with anatomical sites (head and neck, trunk, upper limbs, and lower limbs). The head/neck site is considered a treatment‐resistant region in AD. In our previous study, tralokinumab treatment for moderate‐to‐severe AD consistently improved lesions on all anatomical sites through 48 weeks [30, 31]. However, the site‐specific effectiveness of tralokinumab beyond 48 weeks has not been well described in real‐world practice.

The aim of this study is to evaluate the 72‐week effectiveness of tralokinumab on four anatomical sites (head/neck, trunk, upper limbs, and lower limbs) in patients with AD in real‐world clinical practice. We assessed site‐specific eczema area and severity index (EASI) trajectories, mean percent reductions from baseline, and achievement rates of EASI 75 and EASI 100 over time.

## 2 Methods

### 2.1 Study Design

A prospective study was conducted from October 2023 to October 2025 in Departments of Dermatology at Nippon Medical School Chiba Hokusoh Hospital and Gunma University. This was a real‐world observational study without a control group or interventions. Japanese patients with moderate‐to‐severe AD were clinically diagnosed by dermatologists according to the Japanese Guideline for Atopic Dermatitis 2021 or 2024 [32], depending on the timing of diagnosis. After comprehensive discussion with physicians, patients selected tralokinumab among systemic treatment options. Tralokinumab was subcutaneously administered to patients with an initial dose of 600 mg, followed by 300 mg every 2 weeks (q2W).

Patients who preferred self‐injection at home did so after receiving instruction from medical staff. In combination with tralokinumab, patients were treated with topical corticosteroid (TCS) of class I–III twice daily during the study period (up to Week 72). However, the actual duration, potency choice, and adherence to TCS were determined at the discretion of treating physicians and patients, and details were not recorded on the topical treatment. Data from some patients in this cohort through Week 48 were previously reported in our earlier studies at Weeks 16, 24, 36, and 48 using the same protocol [27, 28, 31, 33, 34, 35, 36, 37, 38, 39, 40, 41, 42]. In our previous site‐specific analysis, detailed evaluation of site‐specific EASI outcomes was limited to Week 36 [31]. Additionally, new patients were enrolled for the current study. This study adhered to the principles of the Declaration of Helsinki (2004) and was approved by the institutional review boards of Gunma University, Nippon Medical School, and Nippon Medical School Chiba Hokusoh Hospital. Written informed consent was obtained from all the participants prior to enrollment.

### 2.2 Data Collection

Patients' background data collected were sex, age, body mass index, duration of AD, and history of allergic rhinitis, bronchial asthma, or allergic conjunctivitis. Data on prior systemic treatments were also collected, including dupilumab (300 mg q2W), lebrikizumab (250 mg q4W), nemolizumab (60 mg q4w), abrocitinib (200 or 100 mg/day), baricitinib (4 or 2 mg/day), upadacitinib (30 or 15 mg/day), cyclosporine A, and systemic corticosteroids. Other baseline assessments included investigator's global assessment, total or site‐specific (head/neck, trunk, upper limbs, and lower limbs) EASI scores, and peak pruritus‐numerical rating scale.

### 2.3 Calculation of EASI Scores and Assessment of Effectiveness on Each Anatomical Site

The EASI score on each anatomical site was calculated by summing up the intensity scores (0–3) of 4 clinical signs × area score (0–6) × proportion of each anatomical site (0.1, 0.2, 0.3, or 0.4 for head/neck, upper limbs, trunk, or lower limbs, respectively).

Total (whole body) EASI and EASI scores on head/neck, trunk, upper limbs, and lower limbs were assessed at Weeks 4, 12, 16, 24, 36, 48, 60, and 72. The proportions of patients achieving EASI 75 (≥ 75% reduction from baseline) and EASI 100 (100% reduction from baseline) were calculated at Weeks 4, 12, 16, 24, 36, 48, 60, and 72 for whole body or each anatomical site. The mean percent reduction of EASI from baseline was also calculated at Weeks 4, 12, 16, 24, 36, 48, 60, and 72 for whole body or each anatomical site.

### 2.4 Inclusion and Exclusion Criteria

All patients had inadequate responses to, or were unsuitable for topical therapy alone. We included patients aged ≥ 15 years with moderate‐to‐severe AD (total EASI ≥ 16 or head/neck EASI ≥ 2.4). Since this was not an interventional study, no wash‐out period was set up when patients were switched from other systemic treatments; the date of switching was defined as baseline (Week 0). Patients who received tralokinumab q4W were excluded because this regimen is not approved in Japan. Additional exclusion criteria were malignancy, stroke, heart failure, myocardial infarction or other severe cardiovascular diseases, active infections such as pneumonia or tuberculosis, known hypersensitivity to tralokinumab or its components, and pregnancy or lactation. We also excluded patients who discontinued and later resumed tralokinumab.

### 2.5 Statistical Analysis

Results are presented as mean ± standard deviation (SD) for normally distributed variables and as median and interquartile range for non‐parametric variables. Correlation was analyzed using Spearman's correlation coefficient. Statistical significance was set at *p* < 0.05. All statistical analyses were performed using Easy R (Saitama Medical Center, Jichi Medical School, Saitama, Japan).

## 3 Results

### 3.1 Baseline Demographic and Disease Characteristics

A total of 208 patients with AD were included, with age mean ± SD 44.7 ± 19.6 years and disease duration 31.9 ± 17.4 years, and male proportion of 67.8% (Table 1). Comorbid conditions included allergic rhinitis (34.1%), bronchial asthma (31.7%), and allergic conjunctivitis (21.2%). At baseline, total EASI was 22.7 ± 9.6. Site‐specific EASI was 1.9 ± 1.5 on head and neck, 7.1 ± 3.5 on trunk, 4.6 ± 2.1 on upper limbs, and 8.9 ± 4.8 on lower limbs.

**TABLE 1:** Baseline demographic and disease characteristics of patients with atopic dermatitis (*n* = 208).

Characteristic	
Male sex, *n* (%)	141 (67.8)
Age (years)^a^	44.7 ± 19.6
Body mass index (kg/m^2^)^a^	22.7 ± 3.8
Disease duration (years)^a^	31.9 ± 17.4
Comorbidities	
Allergic conjunctivitis, *n* (%)	44 (21.2)
Allergic rhinitis, *n* (%)	71 (34.1)
Bronchial asthma, *n* (%)	66 (31.7)
Previous systemic therapy	103 (49.5)
Dupilumab, *n* (%)	23 (11.1)
Nemolizumab, *n* (%)	3 (1.4)
Lebrikizumab, *n* (%)	5 (2.4)
Abrocitinib 200 mg, *n* (%)	1 (0.5)
Abrocitinib 100 mg, *n* (%)	1 (0.5)
Upadacitinib 30 mg, *n* (%)	17 (8.2)
Upadacitinib 15 mg, *n* (%)	38 (18.3)
Baricitinib 4 mg, *n* (%)	8 (3.8)
Baricitinib 2 mg, *n* (%)	4 (1.9)
Corticosteroids, *n* (%)	12 (5.8)
Cyclosporine A, *n* (%)	6 (2.9)
Clinical indexes	
Total EASI^a^	22.7 ± 9.6
EASI on head and neck^a^	1.9 ± 1.5
EASI on trunk^a^	7.1 ± 3.5
EASI on upper limbs^a^	4.6 ± 2.1
EASI on lower limbs^a^	8.9 ± 4.8
IGA, *n* (%)	
Mild (score of 2)	22
Moderate (score of 3)	128
Severe (score of 4)	61
PP‐NRS^a^	7.0 ± 2.7

Abbreviations: EASI, eczema area and severity index; IGA, investigator's global assessment; PP‐NRS, peak pruritus numerical rating scale.

^a^ Data provided as the mean ± standard deviation.

### 3.2 Correlations of Baseline Total EASI With Baseline EASI on Each Anatomical Site

Baseline total EASI score positively correlated with those on all four anatomical sites (Table 2). The results indicate that the severity of rash on each anatomical site might contribute to that on whole body; the strength of association might be rather lower for head and neck (Rho = 0.373) compared to trunk (Rho = 0.796), upper limbs (Rho = 0.81), and lower limbs (Rho = 0.874).

**TABLE 2:** Correlation between baseline total eczema area and severity index (EASI) versus baseline EASI on each anatomical site in patients with atopic dermatitis (*n* = 208).

	Rho	*p*
EASI on head and neck	0.373	< 0.01**
EASI on trunk	0.796	< 0.01**
EASI on upper limbs	0.81	< 0.01**
EASI on lower limbs	0.874	< 0.01**

*Note:* Correlations between variables were examined using Spearman's correlation coefficient.

** Statistically significant at *p* < 0.01.

### 3.3 The Transition of EASI Scores on Different Anatomical Sites During 72‐Week Treatment With Tralokinumab

**FIGURE 1:**
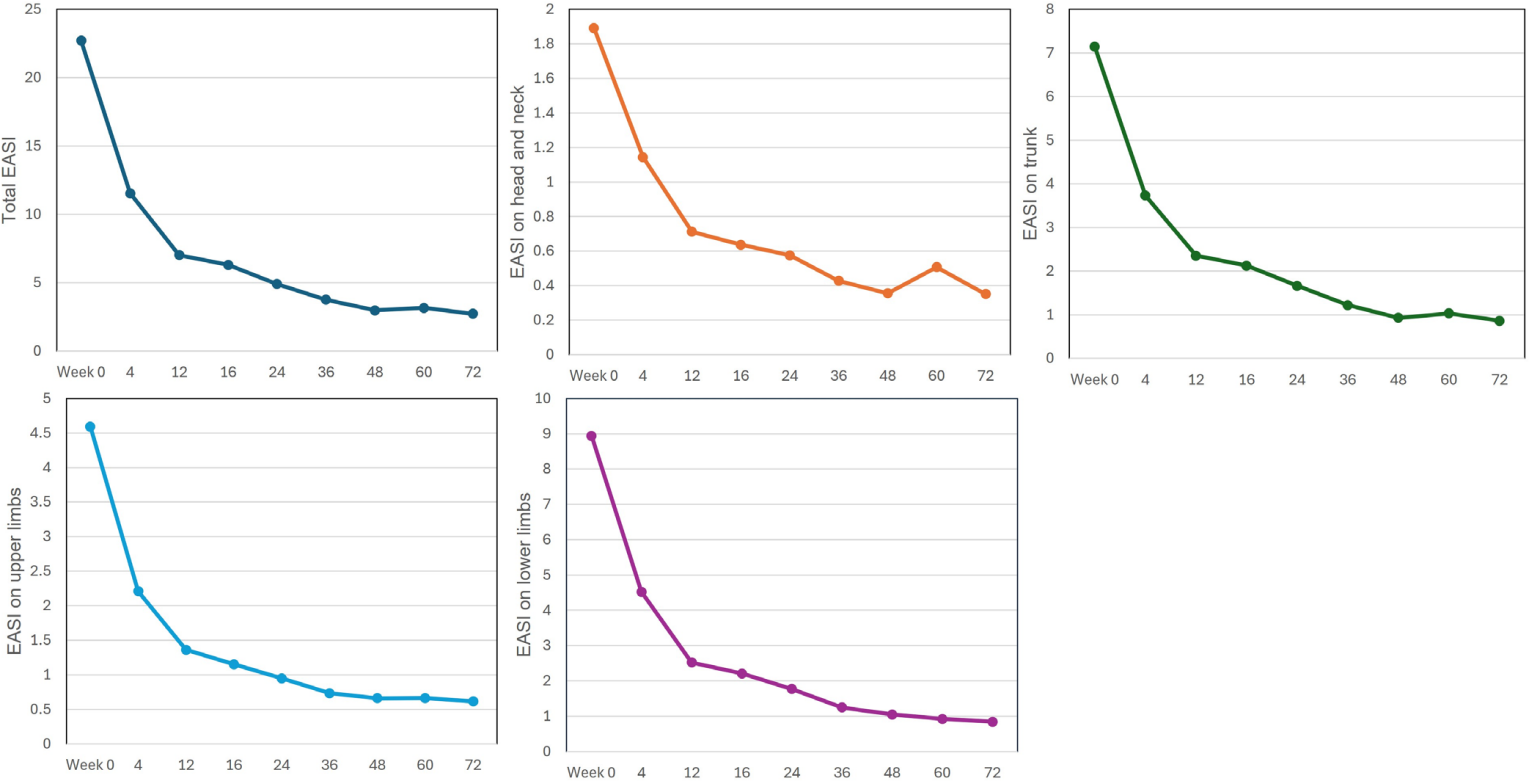
The transition of eczema area and severity index (EASI) on four anatomical sites in patients with atopic dermatitis (AD) (*n* = 208) during 72‐week treatment with tralokinumab. Values of total and site‐specific EASI (head and neck, trunk, upper limbs, and lower limbs) are shown as means at Weeks 0, 4, 12, 16, 24, 36, 48, 60, and 72.

Tralokinumab consistently decreased EASI scores on all anatomical sites, head and neck, trunk, upper and lower limbs, in parallel to total EASI from Week 4 through Week 72 (Figure 1).

### 3.4 The Percent Reduction of EASI on Different Anatomical Sites During 72‐Week Treatment With Tralokinumab

**FIGURE 2:**
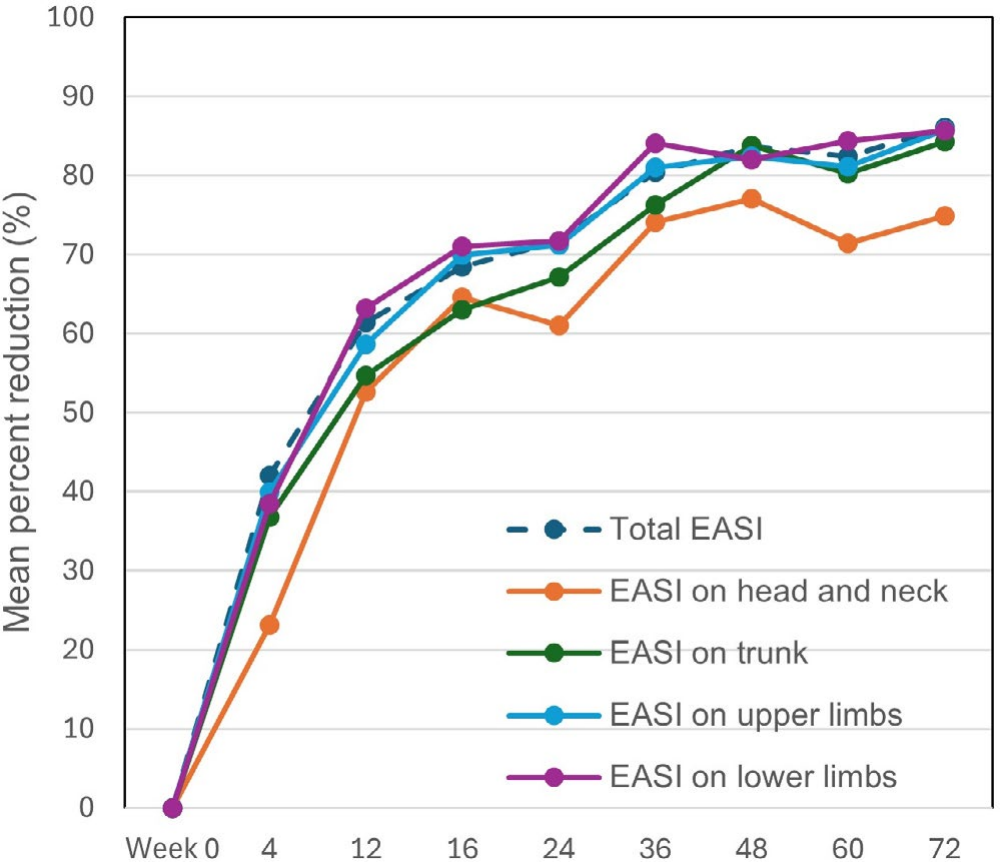
Mean percent reductions of eczema area and severity index (EASI) on four anatomical sites in patients with atopic dermatitis (AD) (*n* = 208) during 72‐week treatment with tralokinumab. The mean percent reduction from baseline is shown for the whole body (total) or head and neck, trunk, upper limbs, and lower limbs at Weeks 0, 4, 12, 16, 24, 36, 48, 60, and 72.

The percent reduction of EASI on each anatomical site increased rapidly at Week 4, and thereafter continued to increase until Week 72, in parallel to that of total EASI (Figure 2). The percent reduction at each time‐point was mostly highest on upper and lower limbs, and lowest on head and neck through 72 weeks. The mean percent reduction of EASI at Week 16 was 64.6%, 63.0%, 69.9%, 71.0%, and 68.4% on head and neck, trunk, upper limbs, lower limbs, and whole body, respectively. At Week 48, the mean percent reduction was 77.1%, 83.8%, 82.4%, 82.1%, and 83.7% on head and neck, trunk, upper limbs, lower limbs, and whole body, respectively. At Week 72, the mean percent reduction was 74.9%, 84.2%, 85.7%, 85.6%, and 86.1% on head and neck, trunk, upper limbs, lower limbs, and whole body, respectively.

### 3.5 The Achievement Rates of EASI 75 and EASI 100 on Different Anatomical Sites During 72‐Week Treatment With Tralokinumab

**FIGURE 3:**
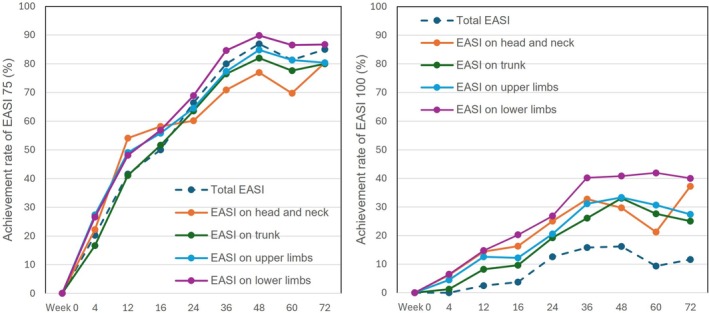
The achievement rates of eczema area and severity index (EASI) 75 and EASI 100 on four anatomical sites in patients with atopic dermatitis (AD) (*n* = 208) during 72‐week treatment with tralokinumab. The achievement rates are shown for the whole body (total) or head and neck, trunk, upper limbs, and lower limbs at Weeks 0, 4, 12, 16, 24, 36, 48, 60, and 72. The left or right panel shows the results for EASI 75 or EASI 100, respectively.

The achievement rate of EASI 75 on each anatomical site as well as whole body gradually increased from Week 4 to Week 72, and the rate at each time‐point appeared mostly highest on lower limbs among anatomical sites; the rate of EASI 75 at Week 72 on head/neck, trunk, upper limbs, lower limbs, and whole body was 80.4%, 80%, 80.3%, 86.7%, and 85%, respectively (Figure 3).

The achievement rate of EASI 100 on each anatomical site as well as the whole body gradually increased from Week 4 to Week 72, and the rate at each time point appeared mostly highest on lower limbs among anatomical sites; the rate of EASI 100 at Week 72 on head/neck, trunk, upper limbs, lower limbs, and whole body was 37.3%, 25.0%, 27.4%, 40.0%, and 11.7%, respectively (Figure 3).

## 4 Discussion

In this study, tralokinumab consistently reduced EASI scores on all anatomical sites through 72 weeks, demonstrating its universal effectiveness regardless of anatomical site. The achievement rate of EASI 75 or EASI 100 at each time‐point through Week 72 appeared slightly higher on lower limbs compared to the other sites; the same trend was seen in our previous study of 36‐week treatment with tralokinumab [31]. Further, treatment with baricitinib 4 mg (Janus kinase [JAK] 1/2 inhibitor) [43] and upadacitinib 15 mg (JAK1 inhibitor) [44] provided slightly higher achievement rates of EASI 75 at Week 12 on lower limbs compared to the other sites. Taken together, tralokinumab, as well as JAK inhibitors, might generate slightly higher therapeutic effects on lower limbs compared to the other sites. On the other hand, another anti‐IL‐13 antibody lebrikizumab provided different site‐dependent effects [45]; Week 36 rates of EASI 75 were mostly similar among four anatomical sites while Week 36 rate of EASI 100 was higher on trunk (46.3%) and upper limbs (48.8%) than on head/neck (28.9%) and lower limbs (33.3%). The reason for the different site‐specificity between two anti‐IL‐13 antibodies is unknown; however, that may be related to differences in treatment period, antibody binding affinity or mechanism of action such as involvement of IL‐13 receptor α2. The site‐dependent effects of anti‐IL‐13 antibodies on AD rash should further be examined over longer treatment periods and in larger cohorts with a variety of ethnicity.

Though percent reductions of EASI on head and neck site were slightly lower compared to the other sites through 72 weeks of tralokinumab treatment, the differences were mostly permissible (Figure 2). Further, EASI 75 and 100 achievement rates on head and neck increased through 72 weeks in the magnitude comparable to those of other sites (Figure 3). These results suggest that tralokinumab can improve AD rash on head/neck to the extent mostly comparable to those of the other anatomical sites, indicating suitability of tralokinumab for this difficult‐to‐treat area.

This study has several limitations. First, the sample size was small. Second, only Japanese patients were included. Third, seasonal flares and poor adherence to topical therapy may have influenced the outcomes. Fourth, the ability to analyze the effectiveness of tralokinumab was limited due to the absence of a randomized control group. Finally, a strict washout period was not set when switching from other systemic therapies to tralokinumab since this was not a clinical trial.

## 5 Conclusions

Treatment with tralokinumab reduced EASI scores across different anatomical sites through 72 weeks. The results suggest that tralokinumab may be consistently effective for AD rash regardless of anatomical site. The achievement rates of EASI 75 and 100 appeared slightly higher on lower limbs while percent reduction of EASI on head and neck appeared slightly lower compared to the other anatomical sites, mostly through 72 weeks.

## Funding

This work is partly supported by 2025 scholarship grants from Maruho Takagi Dermatology Foundation and Torii Pharmaceutical Co. Ltd.

## Ethics Statement

This study was conducted in accordance with the Declaration of Helsinki, and approved by the Ethics Committee of Gunma University, Nippon Medical School Chiba Hokusoh Hospital, and Nippon Medical school.

## Conflicts of Interest

Hidehisa Saeki, Teppei Hagino, Sei‐ichiro Motegi, and Naoko Kanda received lecture fees from LEO Pharma. The other authors have no conflicts of interest to be disclosed. H.S. is an Editorial Board member of Journal of Dermatology and a co‐author of this article. To minimize bias, he should be excluded from all editorial decision‐making related to the acceptance of this article for publication.

## Data Availability

The datasets used and/or analyzed during the current study are available from the corresponding author upon reasonable request.
